# Draft Genome Sequence of *Turicella otitidis* TD1, Isolated from a Patient with Bacteremia

**DOI:** 10.1128/genomeA.01060-15

**Published:** 2015-09-17

**Authors:** Alexander L. Greninger, Varvara Kozyreva, Chau-Linda Truong, Margot Graves, Vishnu Chaturvedi

**Affiliations:** Microbial Diseases Laboratory, California Department of Public Health, Richmond, California, USA

## Abstract

We report the draft genome sequence of *Turicella otitidis* strain TD1, isolated from a central line catheter sample from a patient with a history of bowel obstruction. It contained several genetic determinants of multidrug-resistant phenotypes such as a cfrA 50S methyltransferase, two major facilitator superfamily–type drug resistance transporters, and a putative beta-lactamase.

## GENOME ANNOUNCEMENT

*Turicella otitidis* is a nonfermenting Gram-positive non-spore-forming rod usually isolated from ear exudates. The bacterium was described originally in 1994 from acute and chronic otitis media and has since been isolated from a posterior auricular abscess, mastoiditis, and blood (1–3). To date, only one case of bacteremia due to *T. otitidis* has ever been reported (3). Its association with otitis media is controversial, as it has been isolated from the external auditory canal of healthy subjects at ratios comparable to those noted when *T. otitidis* otitis media was diagnosed (4–6). Most isolates demonstrate high resistance to macrolides and lincosamides with associated 23S rRNA mutations (4, 7).

As only one whole genome of *T. otitidis* had been sequenced previously, we sequenced the draft genome of *T. otitidis* strain TD1, isolated from a central line catheter tip culture. The isolate, and another one on the same patient, from the same source, that was submitted 3 months before, had previously been identified in our laboratory by biochemical and cellular fatty acid analysis.

DNA from strain TD1 was extracted using the Promega Genomic DNA Wizard kit, and paired-end libraries were constructed using the Nextera XT kit. Sequences were adapted and quality (Q30) trimmed using Cutadapt, *de novo* assembled using SPAdes version 3.5, metagenomically screened with SURPI, and annotated via Prokka version 1.1, as described previously (8–12). A total of 739, 492 paired-end reads with an average length of 173 nucleotides were recovered after trimming. *De novo* assembly yielded 160 contigs covering a total of 2,150,112 bp with an *N*_50_ of 24,176 bp, a GC content of 71.2%, an average coverage of 54×, and a total of 1,866 annotated coding sequences.

BLASTn of the assembled 16S sequence from strain TD1 against the NCBI WGS database aligned 99.35% to *T. otiditis* ATCC 51513 and had an average nucleotide identity of 98.75% to ATCC 51513, confirming the identity of the strain (13). Direct mapping of TD1 reads to ATCC 51513 revealed 20,176 variants between the two strains. *De novo* assembly of reads that failed to map to ATCC 51513 revealed a total of 85.4 kb unique sequence, including a locus of 50 kb containing >60 hypothetical protein-coding sequences with no nucleotide homology to the NT database that aligned <35% amino acid by BLASTx and HHPred to various mycobacterium phage protein sequences (14). Genes in the remaining loci not present in ATCC 51513 included an arylsulfatase, a cadmium-cobalt antiporter, a phosphate/phosphonate transporter operon, and a unique ABC transporter, with all predicted proteins having <65% amino acid identity to the closest species, *Corynebacterium glycinophilum* AJ 3170. Antibiotic resistance genes annotated by the Comprehensive Antibiotic Resistance Database included a cfrA 50S methyltransferase (99% amino acid to *T. otitidis* ATCC 51513) and two major facilitator superfamily–type drug-resistance transporters (99% amino acid *T. otitidis* ATCC 51513 and 57 to 59% amino acid to *Corynebacterium* spp.) (15). In addition, a putative beta-lactamase aligned 93% by amino acid to *T. otitidis* ATCC 51513.

### Nucleotide sequence accession number.

This whole-genome shotgun project has been deposited at DDBJ/EMBL/GenBank under the accession number LBNF00000000.
